# Farmers’ Economic Status and Satisfaction with Homestead Withdrawal Policy: Expectation and Perceived Value

**DOI:** 10.3390/ijerph17197110

**Published:** 2020-09-28

**Authors:** Yaoyang Zhao, Scott Cloutier, Hongqing Li

**Affiliations:** 1 School of Public Administration, Hohai University, Nanjing 211100, China; lihongqing163@126.com; 2 Julie Ann Wrigley Global Institute of Sustainability, School of Sustainability, Arizona State University, Tempe, AZ 85281, USA; scloutie@asu.edu

**Keywords:** homestead withdrawal, policy satisfaction, influencing factors, structural equation modeling

## Abstract

The withdrawal of homesteads is an effective way to improve the efficiency of rural construction land use and is being piloted in many regions of China, but the mechanism influencing farmers’ satisfaction with the withdrawal policy is unclear. This paper aimed to investigate the relationships among farmers’ economic status (ES), policy expectation (PE), policy perceived value (PPV), and farmers’ satisfaction with homestead withdrawal policy (policy satisfaction; PS). The study examined the mediating effects of PE and PPV on the relationship between ES and PS. The data obtained from a questionnaire of 287 households in Jinhu County, Jiangsu Province, China. After surviving from reliability and validity tests, a structural equation modeling (SEM) with latent variables was specified and estimated using Mplus. From the study results, we found significant positive relationships between ES, PP, and PS, but significant negative relationships between ES, PE, PPV, and PS. Also, our research found PE and PPV as potential mediators on ES-PS relation. We propose recommendations from three aspects to improve farmers’ satisfaction with future implementations of the homestead withdrawal policy. Our results provided new insights into how to improve the performance of homestead withdrawal policy.

## 1. Introduction

Over the past 40 years, the urbanization level of China has increased dramatically, from 17.9% in 1978 to 60.6% in 2019. Accompanied with rapid urbanization, more than 250 million rural people have left their lands and villages to start new lives in factories and cities [1,2]. However, with the advancement of the urbanization process, the rural homestead utilization area does not appear to be decreasing as initially expected. The reverse-evolution pattern of “population reduction and construction land increase” has appeared in various regions [3]. According to statistics, the total rural resident population of China is decreasing at a rate of 1.6% each year. However, the area of homesteads in rural areas is increasing by more than 1,000,000 km^2^ per year, with an annual growth rate of 1% [4]. Paradoxically, although the total area of rural homesteads in China has been expanding, the phenomenon of idle and abandoned housing sites such as “empty houses” and “hollow villages” has become more frequent and continues intensifying the trend [5]. Statistics show Chinas’ idle homesteads in rural areas amount to three million hm^2^, with nearly six million hm^2^ in low-efficiency homesteads, while the vacancy rate of rural homesteads has reached 10.7% [6]. Therefore, how to effectively utilize idle rural homesteads has become a pressing issue.

To solve this dilemma, in 2005, the Chinese government promulgated the land use policy of “Linking the Increase in Urban Construction Land with the Decrease in Rural Construction Land” to explore the way of “withdrawal of homesteads” [7].

Homestead withdrawal includes both homestead-centered withdrawal mechanisms and compensation and guarantee mechanisms [8]. It offers an effective solution to the issue of extensive use of rural construction land, and it has received growing attention from many local governments [9]. Some typical withdrawal modes have formed based on regional characteristics, such as exchanging a homestead for an apartment, double abandonment (DA) and a land ticket transaction [1]. As of the end of 2018, 140,000 households had withdrawn from homesteads in 33 pilot areas across the country, with a total area of 56 km^2^ [10].

However, the homestead withdrawal mechanism has a series of problems, such as vague property rights, lack of fixed-term paid-use systems, and defects in the conditions for the withdrawal of homesteads [11]. This leads to the lower willingness of farmers to withdraw homestead and the area of rural homesteads continuing to expand [12,13]. Furthermore, some reports have said the homestead withdrawal policy meets with resistance from villagers in some areas, for a variety of reasons [14]. So, it is still unclear what is the best homestead withdrawal mode, not only for farmers and but also for society. There has not been a well-recognized mode that can be taken for replication across the country.

Research on withdrawal from rural homesteads has become a hot topic. Over the last ten years, much research has focused on the following areas: (1) driving forces in homesteads’ withdrawal [15,16]; (2) farmers’ willingness to withdraw from homesteads [17,18]; (3) compensation for homestead withdrawal [19]; (4) working mechanism of homestead withdrawal [20]. These studies provide abundant theoretical guidance for continued research.

In terms of farmers’ policy satisfaction (PS), some studies have tried to construct index systems to evaluate the satisfaction with compensation rates [21,22], settlement communities [23], and living environments [24,25] in the process of homesteads withdrawal. Zhao and Zhang (2013), in the case of Chengdu City, found an enormous gap between farmers’ expectations of the policy and the results of its implementation [26]. Hu Yingen (2018) discovered that the more farmers’ actual living standards was improved after homestead withdrawal, the higher their satisfaction with the policy was [27].

Li Min (2019) indicated that family resource endowment is a vital antecedent factor affecting farmers’ satisfaction, and perceived quality and perceived value (PV) are the most critical factors affecting farmers’ satisfaction [28]. Liu et al. (2020) have found that the better the economic status (ES) of a rural household, the stronger its members’ perception of the benefits of increased economic income; the lower the impact of the rising cost of living on their lives, and the higher their satisfaction with the ES after the homestead withdrawal [29].

From this review of previous research, although some attention has focused on farmers’ satisfaction with the homestead withdrawal policy, existing studies differ in the selection of factors influencing farmers’ satisfaction. They do not construct a complete and unified analytical framework. Moreover, the research methods are mostly limited to Analytic Hierarchy Process (AHP), fuzzy evaluation and logistic regression model. These cannot reflect the formation mechanism of farmers’ satisfaction and its influencing mechanism, thus they fail to clarify the various interrelated influencing factors.

In summary, the Chinese government must urgently design and apply a national model for effective withdrawal of homesteads. Farmers, as the actual occupants and users of homesteads, are not only the beneficiaries of homestead withdrawal policy, but also the direct participants and key stakeholders in homestead withdrawal activity [30]. Only when farmers are satisfied with the homestead withdrawal policy will more rural households be willing to withdraw from their homesteads in the future.

Therefore, a scientific method that measures farmers’ satisfaction with the policy should be proposed after the implementation of the homestead withdrawal policy. Also, factors that may affect farmers’ satisfaction must be studied. This will not only help improve the implementation performance of the policy, but also plays an important role in advancing Chinas’ national reform of the rural homestead management system.

Given the aforementioned problems, our present study aims to (1) comprehensively analyze the relationships among farmers’ ES, policy expectation (PE), policy perceived value (PPV), and farmers’ satisfaction with homestead withdrawal policy (PS) in the context of homestead withdrawal. It also aims to (2) identify the mediating effects of PE and PPV on the relationship between farmers’ ES with PS and to (3) promote suggestions on effectively implementing the homestead withdrawal policy.

The remainder of this paper is organized as follows. Section 2 presents the theory, hypotheses, and model employed in this study. Section 3 describes the study area, questionnaire, and research methodology. Section 4 elaborates the key results of the study. Discussion and implication, and conclusion are provided in Section 5 and Section 6, respectively.

## 2. Theory Hypotheses and Model

### 2.1. Theory and Research Hypotheses

Satisfaction is a subjective psychological evaluation of pleasure and happiness when something meets peoples’ expectations. Farmer’ satisfaction with homestead withdrawal policy is the quantitative value of the psychological evaluation of farmers’ living and livelihood after homestead withdrawal. Measuring satisfaction requires establishing an appropriate model. The most widely used type of model is the customer satisfaction index. Such models include the Swedish Customer Satisfaction Barometer, the American Customer Satisfaction Index, the European Customer Satisfaction Index, and the China Customer Service Index [31].

In recent years, customer satisfaction theory has been used widely in evaluating the performance of public policies [32,33,34,35]. In this paper, we will regard farmers who withdraw from their homesteads as customers, and the homestead withdrawal policy as a product provided by the government. Based on the framework for measuring and the comparison of customer satisfaction, proposed by Johnson and Fornell [36], we retain individual differences, customer expectations, and PV as antecedent variables of satisfaction in the model.

Because homestead withdrawal is a one-time policy activity, there is no subsequent consumer behavior, such as customer loyalty or complaints, so we eliminated behavioral consequences as posterior variables. We used this model to analyze the main factors, and their influence paths, regarding farmers’ satisfaction with the homestead withdrawal policy.

1. Relationship between farmers’ economic status and policy satisfaction.

Individuals have their own opinions when it comes to satisfaction with customer service. For the farmers in the studied areas, the greatest differences among them depend on familie’s relative ES. The ES of a rural family can reflect how difficult it is for the family to change its conditions, and it can affect whether the family is willing to withdraw from its homestead. Studies have shown that farmers’ ES is closely related to their satisfaction. Zhang et al. (2011) indicated that the age of the head of household, number of laborers in the family, and family income, each plays a role in farmer’s satisfaction [37]. Lian et al. (2015) found that family characteristics affect the health and life satisfaction of the parents whose children are migrant workers [38]. Luo and Timothy (2017) found that farmers’ education level, employment characteristics, and family size have a significant impact on the satisfaction of farmers involved in land consolidation [39]. Gao et al. (2017) identified objective factors, such as farmers’ household characteristics, that also significantly influenced farmer’s satisfaction with the land titling process [40]. Cao et al. (2017) pointed out that household factors, such as per-capita household income and source of income, have significant effects on the satisfaction of poverty alleviation policies among farmers in Shaanxi, showing a weak positive correlation [41].

The withdrawal of homesteads has relatively little impact on high-income families, but it has a significant impact on families with a low income [42]. When a households’ predominantly agricultural income moves to a house far away from the contracted land, it has an adverse impact on agricultural production. On the contrary, it will have almost no impact if the household can successfully transfer out of the contracted, cultivated land after homestead withdrawal, and this would increase the familys’ satisfaction to some extent. So, in terms of homestead withdrawal, we assume families of varying economic status have varying experiences of satisfaction with the policy. In the case of households whose income is based on agriculture, if they move to a house far from the contracted land after homestead withdrawal, there will be a negative impact on agricultural production due to the increased farming radius. However, for households based on nonagricultural income, the withdrawal of homesteads has little effect in this regard. Accordingly, we propose the following hypothesis:

**Hypothesis 1** (**H1**)**.**  *Family economic status has a positive effect on farmers’ PS*.

2 Relationship between policy expectation and policy satisfaction

Expectation is the category of mental activity that refers to the goals or benefits of people, based on their experiences, wish to achieve or obtain in a given situation. A review of the literaturesuggests that expectation plays a major role in satisfaction. In cognitive models of satisfaction [43], Oliver (1980) suggested that a consumers’ satisfaction with a product or a service depends on the quality actually experienced, and on the expected quality [44]. Tversky and Kahneman (1991) also indicated that consumers’ utility functions (goal attainment or want satisfaction) are dependent on a reference level. When expectation becomes the reference level with which an actual consumption experience is compared, consumers will be satisfied if they receive the expected quality, and otherwise they will be dissatisfied [45].

According to previous studies, the issues that farmers are most concerned about before withdrawing from their homesteads are the compensation they may obtain, the standard of living they may later enjoy, and the existence of a sound follow-up guarantee [46]. How their expectations will be met becomes the standard by which they judge whether the policy is satisfactory, so PE has an essential impact on satisfaction. It seemed reasonable for us to expect that the greater farmers’ PE, the lower their level of satisfaction. Farmers with lower PE are more likely to fulfill their expectations, so that they will be more satisfied. Accordingly, we propose the following hypothesis:

**Hypothesis 2** (**H2**)**.**  *Policy expectation has a negative effect on farmers’ PS*.

3 Relationship between PPV and PS

PV has been measured as both customer utility and as the ratio of perceived benefits relative to sacrifice, quality, worth, and so forth [47]. From a behavioral perspective, PV is defined as “the consumers’ overall assessment of the utility of a product (or service) based on perceptions of what is received and what is given” [48]. As to the utilitarian perspective, it is a function of acquisition utility and transaction utility [49]. Previous research has suggested that PV is an essential antecedent to satisfaction [50,51,52].

Farmers’ PPV of homestead withdrawal policy refers to subjective feelings about whether it is worthwhile, as formed by farmers comparing situations before and after withdrawal from their homesteads. So, when farmers in the process of homestead withdrawal feel more deeply that they have experienced improvement in housing conditions, employment, income, other living conditions, and social security, their satisfaction with the policy will be relatively high. On the contrary, farmers are bound to be less satisfied if they cannot feel the value of the policy, such as when they assess changes in their living environments, when the policy brings little benefit, or when benefits are lost. Therefore, PPV has a direct impact on farmers’ satisfaction during homestead withdrawal. Accordingly, we propose the following hypothesis.

**Hypothesis 3** (**H3**)**.**  *The policy’s perceived value has a positive effect on farmers’ PS*.

4 Relationships among ES, PE, and PPV

PV is a subjective, dynamic construct that varies across clients [53]. The family ES of Chinese farmers is closely related to education levels and ideological attitudes. Farmers with poorer family economic conditions (because of their poor understanding of the policy and the influence of news reports of urban construction projects that produce overnight riches from demolition) may have unrealistically high expectations of the homestead withdrawal policy. However, for families with better ES, there is usually a more rational expectation with regard to the homestead withdrawal policy. Therefore, we assumed that farmers’ ES will affect their perception of PV.

Also, the ES of a household will also affect policy perception. Chen and Li (2012) stated that people at different income levels have different subjective perceptions of life [54]. Farmers with lower family economic conditions, due to these conditions, have relatively poor perceptions of the policy implementation process and results. Farmers with better family economic conditions can experience some slight changes around them and feel the changes brought about by the implementation of the policy in all aspects.

Furthermore, PPV, as the outcome variable of farmers’ PE, is closely related to PE. In general, the higher farmers’ PE, the lower their PPV. This is because overall dissatisfaction with the policy will cause farmers to turn a blind eye to the tangible results of implementing the policy. In contrast, if farmers’ original PE is relatively low, they can objectively perceive the slight changes brought about by implementing the policy.

Accordingly, we made the following predictions:

**Hypothesis 4** (**H4**)**.**  *Family ES has a negative effect on farmers’ PE*.

**Hypothesis 5** (**H5**)**.**  *Family ES has a positive effect on farmers’ PPV*.

**Hypothesis 6** (**H6**)**.**  *PE has a negative effect on PPV*.

### 2.2. Conceptual Model

**Figure 1:**
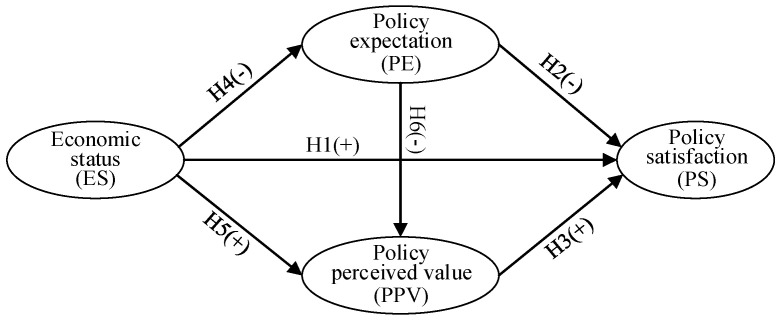
Theoretical framework: Study and hypotheses.

Based on the above theoretical analysis, we constructed a conceptual model analyzing the relationship between ES, PE, PPV, and PS (Figure 1). In the model, ES, PE, PPV, and PS are all latent variables that cannot be directly measured and need to be measured using observation variables. The model describes the causal relationship between latent variables. The exogenous latent variable is ES, and the endogenous latent variables include PE, PPV, and PS.

## 3. Methodology

### 3.1. Study Area

**Figure 2:**
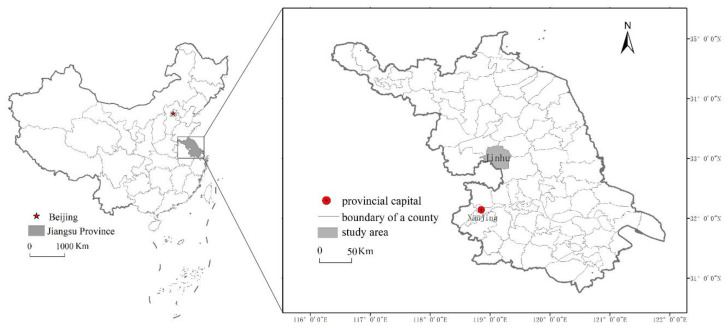
Location of the study area.

As a case study, we took Jinghu County, located in central Jiangsu Province, East China (Figure 2). In 2018, the population is approximately 349,000 people. The gross agricultural output value of Jinghu is about CNY 3.7 billion (USD 530 million), which accounts for just 12.5% of local GDP. The total area of the county is 1,393.86 km^2^, of which 801.43 km^2^ is land area, accounting for 57.5% of the total area; 446.96 km^2^ consists of water surface, and 145.47 km^2^ of tidal flats, with water surface and tidal flats accounting for 42.5%.

In recent years, with the rapid development of the local economy, a large number of rural households have moved to the cities, joining growing numbers of migrant workers. Conversely, despite rapid urbanization, the rural building land of Jinhu County is continually increasing. Statistical data show that the countys’ rural population decreased from 269,600 in 2000 to 248,600 in 2018; the amount of rural building land rose from 106.19 km^2^ to 136.07 km^2^ during the same period. Thus, the result is that the per-capita rural building land area increased from 393.87 m^2^ to 547.34 m^2^. Due to the fact that a large number of rural households actually live in cities, many vacant houses and idle lands have appeared in rural areas of Jinhu County—more than 60% of rural housing in the county has become idle on a year-round basis. To solve the increasingly severe conflict of “eating” issues versus “construction” problems, in 2016, Jinhu County undertook a national pilot project of “Paid Withdrawal of Homestead”. In 2018, Jiangsu Province additionally chose Jinhu County as a recent example of homestead reform pilot zones. Therefore, Jinghu County provides a typical case for study of homestead withdrawal policy.

### 3.2. Questionnaire and Data Collection

The survey was carried out in January 2019. The questionnaire covered four villages of Fulian, Yuguo, Xinsheng, and Heying, encompassing a wide range of regional representation for Jinhu County. The members of the survey group were trained to obtain relevant information from the interviewees through face-to-face interviews. A total of 318 questionnaires were distributed, and 287 valid questionnaires were obtained after eliminating information loss and errors.

The content of the questionnaire included (1) household ES about homestead area of family, annual net income of the family, and percentage of non-agriculture income. (2) farmers’ PE about overall policy expectations, the expectation of compensation rate, and the expectation of the quality of life. (3) farmers’ PPV about changes in employment, income, the living environment, and social security., and (4) farmers’ PS about overall satisfaction with the policy, satisfaction compared to expectations, and satisfaction compared to other land acquisition policies. A five-point Likert scale was used for the questionnaire (1 = very unsatisfied; 2 = unsatisfied; 3 = moderate; 4 = satisfied; and 5 = very satisfied). The detailed information of all investigated objects is listed below in Table 1.

### 3.3. Structural Equation Modeling

Structural Equation Modelling (SEM) was applied to investigate the factors affecting farmer’s satisfaction with the homestead withdrawal policy. The model included four latent variables, ES, PE, PPV, and PS. First, Confirmatory Factor Analysis (CFA) was used to measure the internal consistency reliability, convergent validity and discriminant validity of the constructs in our proposed model. Then, SEM method was applied to quantify the strength of the interaction between the four latent variables.

## 4. Results

### 4.1. Demographic Information

The demographic characteristics of the interviewed farmers appear in Table 2. Amongst the respondents, 63% were male and 37% were female, and the average age was 44. The educational level of the respondents was low, with the average as middle school(Chinas’ nine-year compulsory education level) but showing respondents’ literacy and capacity to understand the homestead withdrawal policy. Moreover, the average family size was 5.453, and the labor size was 3.107. The surveyed families owned an average of 4.7 Mu (3135 m^2^) of cultivated land and 206 m^2^ of the homestead. The per capita annual income was CNY 9854 (USD 1407).

### 4.2. Measurement Model

Table 3 shows the result of CFA analysis on the four constructs of EC, PE, PPV, and PS. It revealed that the composite reliability (CR) of each construct ranged from 832 to 896, exceeding the 70 CR threshold value, and giving evidence of internal consistency reliability [55]. Also, the range of factor loadings for ES, PE, PPV, and PS are 0.691–0.827, 0.727–0.841, 0.796–0.842, and 0.843–0.877, respectively, exceeding the accepted criterion of 0.60 [56]. It shows preliminary evidence for the convergent validity of the measurement model. Meanwhile, the average variance extracted (AVE) of all constructs ranged from 0.556 to 0.745, exceeding the 0.50 AVE threshold value [57], and thus the convergent validity was acceptable.

Discriminant validity refers to the extent to which measures do not reflect those for other variables; it is indicated by low correlations among the measure of interest and the measures for other constructs. Table 4 shows that correlation values between ES and PPV (r = 0.472;*p* < 0.01), between ES and PS (r = 0.632; *p* < 0.01), and between PPV and PS (r = 0.759; *p* < 0.01) were statistically significant and positive. Meanwhile, the correlation values between ES and PE (r = −0.294; *p* < 0.01), between PE and PPV (r = −0.642; *p* < 0.01), and between PE and PS (r = −0.602; *p* < 0.01) were statistically significant and negative. Bold values in Table 4 show the variables’ discriminant validities. The discriminant validities for ES, PE, PPV, and PS were 0.746, 0.790, 0.826, and 0.863, respectively. All these values are the square root of the average variance extracted, and they are greater than the values of the constructs’ intercorrelations.

### 4.3. Hypothesis Testing

The hypothesized model was analyzed using SEM with maximum likelihood estimation in Mplus [58]. A bootstrapping procedure was conducted with 1000 iterations, to examine the statistical significance of the weights of subconstructs and of the path coefficients. We used χ^2^/df, the comparative fit index (CFI), the Tucker–Lewis index (TLI), the root mean square error of approximation (RMSEA), and the standardized root mean square residual (SRMR) as the fit indices during the CFA analysis where χ^2^/df should be <3 [59], values of CFI and TLI should be ≥0.90 [60], and RMSEA and SRMRshould be <0.08 [61]. The overall model fit of the structural equation modeling provided satisfying results. The values included χ^2^/df = 1.067, CFI = 0.998, TLI = 0.997, RMSEA = 0.015, and SRMR = 0.03.

Hypothesis 1 stated, “Family ES has a positive effect on farmers’ PS”. As shown in Table 5, we found hypothesis 1 significant and positive because the regression (β =0.355; t = 6.649; *p* < 0.001) provided supportive evidence. Hypothesis 2 stated, “PE has a negative effect on PS ”. The regression estimates (β = −0.201; t = −3.020; *p* < 0.01) provided sufficient support for hypothesis 2. Hypothesis 3 stated, “PPV has a positive effect on farmers’ PS”. The regression (β = 0.463; t = 6.911; *p* < 0.001) provided enough support for hypothesis 3. Hypothesis 4 stated, “Family ES has a negative effect on farmers’ PE”. We found hypothesis 4 significant and negative because the regression (β = −0.294; t = −4.667; *p* < 0.001) provided supportive evidence. Hypothesis 5 stated, “Family ES has a positive effect on farmers’ PPV”. The regression (β = 0.310; t = 5.375; *p* < 0.001) provided sufficient support for hypothesis 5. Hypothesis 6 stated, “PE has a negative effect on PPV”. The regression estimates (β = −0.551; t = −10.666; *p* < 0.001) provided sufficient support for hypothesis 6. We also measured a 95% confidence interval for Table 5.

In addition, we estimated the indirect effects of PE and PPV, as mediating variables, on ES and PS. We performed bootstrapping at a 95% confidence interval using 1000 boot samples, and we calculated the confidence intervals as well as z values for the lower and upper bounds. The bootstrapping analysis for indirect effects is exhibited in Table 6, where we found significant mediating effects for PE on the ES→PS relationship (β = 0.059; z = 2.632, and *p* = 0.008), for PPV on the ES→PS relationship (β = 0.143; z = 4.317, and *p* < 0.001), for PE and policy perception on the ES→PS relationship (β = 0.075; z = 3.513, and *p* < 0.001), for PPV on the PE→PS relationship (β = −0.025; z = −5.305, and *p* < 0.001), and for PE on the ES→PPV relationship (β = 0.162; z = 4.417, and *p* < 0.001). These results provided additional support for Hypothesis 1, 2, and 5.

## 5. Discussion and Implications

### 5.1. Discussion

The present research used confirmatory factor analysis and structural equation modeling techniques to investigate and validate the structural relationships between farmers’ ES, PE, and PPV with PS. The purpose of the research was to investigate the impact of family ES on farmer’s PS by way of PE and PPV, and then get considerable insights on how PS can be enhanced. To the best of our knowledge, no previous research has been conducted to evaluate the influence of ES on farmers’ PS through PE and PPV.

First among the interesting insights we have gained from this study, the results shown in Table 5 and Table 6 revealed that farmers’ ES has a significant and positive effect on PS, with total factor loading at 0.632 including direct effect (β = 0.355; *p* < 0.001), through PE (β = 0.059; *p* = 0.012), through PPV (β = 0.143; *p* < 0.001), and through PE and PPV (β = 0.075; *p* < 0.001). This outcome supports previous studies’ findings that showed ES as a key factor with a positive effect on linking PS and influencing farmers’ willing withdrawal from homesteads [62]. This is because ES is related to farmers’ education, ideology, and comprehensive quality. Farmers with high ES usually understand the homestead withdrawal policy well, while farmers with lower ES usually have high PE, looking forward to overnight riches through homestead withdrawal. As a result, families’ ES shows a negative correlation with PE. Table 5 also shows a direct impact of household ES on PPV at 0.310, indicating that farmers with high household ES are likely to have learned more about the policy and to participate in the public discussion on process in implementing policy (because of their relatively high overall quality). They are thus better able to perceive the value of the policy, such as the effect of village construction and so on.

Second, PE has a negative impact on PS in homestead withdrawal. However, the overall impact is relatively smaller than ES, with a standardized path coefficient of −0.456, where the direct impact is −0.201 and the indirect impact through PPV is −0.255, and the model results are consistent with the original hypothesis. This indicates that the higher the PE (i.e., the higher the expectation of the compensation standard and other supporting measures), the better the quality of life and income after homestead withdrawal. However, if the actual benefits brought about by the policy are lower than expectations, a huge psychological gap will lead to farmers’ lower PS. Therefore, the final results of the model show that PE is negatively correlated with PS.

Third, PE has a strongly negative relationship with PPV. This result is consistent with our Hypothesis 6 in our study, that higher PE leads to lower PPV. The investigation of our study also showed that the PE of most farmers in the study area is realistic, as they only hope that they can use the compensation to buy a relatively good residence after homestead withdrawal. They also clearly realize that it is impossible to achieve a radical change in their lives and income through the policy. As a result, those farmers who had a relatively strong understanding of policies had more reasonable PE, and correspondingly their PPV were higher.

Fourth, PPV has a direct positive impact on PS, with a standardized path coefficient of 0.463, indicating that it has a significant impact on PS. This means the greater the improvement of farmers’ living conditions after homestead withdrawal (e.g., employment, income, living environment, and social security), the greater their satisfaction with the policy.

Finally, it is essential to note that the direct effect of PPV on PS (β = 0.463) is significantly higher than PE on PS (β = −0.201). Also, the indirect effect of ES on PS through PPV (β = 0.143) is also higher than that through PE(β = 0.059). It means that as a mediator variable, PPV has a greater effect on PS than does PE.

### 5.2. Practical Implications

This study investigated farmers’ satisfaction with homestead withdrawal policy and, through structural equation modeling analysis, identified the significant contributing factors, namely farmers’ ES before homestead withdrawal, PE, and PPV. The findings of the present study offer empirical evidence of how satisfaction with the homestead withdrawal policy is affected and can be enhanced. In line with these findings, we present some policy recommendations from three aspects, to improve farmers’ satisfaction levels in future implementations of homestead withdrawal policy in other parts of China.

1 Select more developed regions to implement the policy

Household ES, through the two mediating variables of PE and PPV, has a positive effect on satisfaction with homestead withdrawal policy. Thus, the greater the ES of households willing to withdraw from their homesteads, the greater their satisfaction after homestead withdrawal. Household ES is measured by four indicators: cultivated land per capita, family homestead area, family annual net income, and percentage of non-agriculture income. Therefore, first, from a location perspective, it is necessary to select more developed regions, such as suburban areas of cities or industrialized areas, where rural households have better economic conditions. Secondly, if an area has established a circulation platform for collectively contracted cultivation land, it will help farmers to transfer contracted farmland easily, and it will allay their worries about engaging full-time in nonagricultural work after homestead withdrawal. Finally, it is essential that there is sufficient local non-farm employment for farmers, a key point for achieving sustainability in their livelihoods.

As to which locations are suitable for pilot studies of homestead withdrawal policy, Zheng and Ding (2013) pointed out that the feasibility of homestead withdrawal depends mainly on villager’s income and local economies [63]. Only places with a high degree of economic development and a small gap between rich and poor rural households are suitable, due to their more comprehensive social security systems [64].

2 Strengthen publicity to lower policy expectations

As farmers’ expectations of homestead withdrawal policy affect their PPV and ultimately their PS, the greater the psychological gap between PE and actual policies, the lower the farmers’ PS. Farmers’ PE is affected mainly by due compensation and their expectation, and can be increased or decreased by external influences, such as through mass media and advertising [65]. Although expectations cannot be controlled, deserved compensation can provide farmers with a clear understanding through policy publicity, and it can prevent farmers from running into the decreased PS resulting from overly high expectations due to their lack of understanding about the policy. Therefore, to eliminate the communication gap and decrease farmers’ PE, publicity and transparency must be improved for the homestead withdrawal policy. It is suggested that the publicity of the homestead withdrawal policy should be strengthened in the future, using media such as community bulletin boards and information service platforms, to enable farmers to better understand, and especially to clarify the difference between land acquisition policy and voluntary homestead withdrawal policy. This way, farmers can correct their understanding of the homestead withdrawal policy, dispel their excessively high PE, and then improve their PS.

3 Enhance public participation to improve policy perception

Farmers’ policy perceptions value have a significant positive impact on PS. PPV is measured by four indicators: change in employment, change in income, change in the living environment, and change in social security. Therefore, to improve farmers’ PPV, several supportive policies should be provided to farmers, including skills training to improve their employment prospects, microfinance to facilitate their transition from agricultural to nonagricultural employment, and social insurance to those willing to withdraw from their contracted farmland simultaneously.

Lisec (2014) indicated that farmers’ active participation affects their perceptions and levels of satisfaction [66]. Therefore, to enhance farmer’s perception of homestead withdrawal policy while strengthening policy publicity to enhance farmers’ understanding, it is also necessary to take farmers as the main body of homestead withdrawal. In the process of guiding farmers to withdraw from their homesteads, local governments should take the needs of farmers as the guidelines, taking the interests of farmers into account and fully respecting the wishes of farmers, while incorporating farmers’ opinions into the public goods and services provided by the local community. However, due to the currently low willingness of Chinese farmers to participate in public affairs and their lack of public spirit [67], it is recommended that local governments optimize the public participation environment and that they play a guiding role to enhance the awareness of farmers’ participation, so as to ultimately achieve a more remarkable improvement of farmers’ perception of homestead withdrawal policy.

## 6. Conclusions

The withdrawal of homesteads is an important way to enhance the efficiency of rural construction land use, and also an effective means of improving the rural living environment. It is currently being piloted in many parts of China. In this study, we proposed a structural model of farmer’s satisfaction with the homestead withdrawal policy. We examined the effects of farmers’ ES and two mediating variables (PE and PPV) on satisfaction with homestead withdrawal policy. The results show that there are significant positive relationships between ES, PP, and PS, but significant negative relationships between ES, PE, PPV, and PS. Family ES not only plays a significant role in directly enhancing farmers’ PS; it also affects satisfaction via PE and PPV. Our research can be helpful to understand what variables affect farmers’ satisfaction in the context of homestead withdrawal. The results suggest that in order to maximize farmer satisfaction, we need to focus not only on farmers’ ES but also on farmers’ PE and PPV. However, some study limitations should be acknowledged. First, several models of homestead withdrawal are being piloted in China, but our study only selected one of them as the object of study. Second, our study only selected farmers in one county as the research sample, and this does not provide good coverage of farmers considering the differences in ES. Therefore, further researches should consider the limitations of this paper, and carry out a comparative analysis on the factors affecting farmer’s satisfaction in areas at different stages of economic development and between different homestead withdrawal modes to improve the reliability and validity of the results.

## Figures and Tables

**Table 1:** Variables used to analyze factors affecting farmers’ satisfaction with homestead withdrawal policy.

Dimensions	Items	Definition
Economic status (Y1)	Cultivated land per capita/X1	1 = [0,0.5], 2 = (0.5,0.8], 3 = (0.8,1], 4 = (1,1.5], 5 = (1.5,+]
	Homestead area of family/X2	1 = [0,100], 2 = (100,200], 3 = (200,300], 4 = (300,400], 5 = (500, +]
	Annual net income of family/X3	1 = [0,10000], 2=(10000,20000], 3 = (20000,30000], 4 = (30000,50000], 5 = (50000, +]
	Percentage of non-agriculture income /X4	1 = [0,10%], 2 = (10%, 30%], 3 = (30%, 60%], 4 = (60%, 80%], 5 = (80%, 100%]
Policy expectations (Y2)	Overall policy expectations/X5	1 = Very low; 2 = Low; 3= moderate; 4 = High; 5 = Very high
	Expectation of compensation rate/X6	1 = Very low; 2 = Low; 3 = moderate; 4 = High; 5 = Very high
	Expectation of quality of life/X7	1 = Very low; 2 = Low; 3 = moderate; 4 =High; 5 = Very high
Policy perceived value(Y3)	Changes in employment/X8	1 = Very little, 2 = Little, 3 = Moderate, 4 = Big, 5 = Great
	Changes in income/X9	1 = Very little, 2 = Little, 3 = Moderate, 4 = Big, 5 = Great
	Changes in the living environment/X10	1 = Very little, 2 = Little, 3 = Moderate, 4 = Big, 5 = Great
	Changes in social security/X11	1 =Very little, 2 = Little, 3 = Moderate, 4 = Big, 5 = Great
Policy satisfaction (Y4)	Overall satisfaction with the policy/X12	1 = Very unsatisfied, 2 = Unsatisfied, 3 = Moderate, 4 = Satisfied, and 5 = Very satisfied
	Satisfaction compared to expectations /X13	1 = Very unsatisfied, 2 = Unsatisfied, 3 = Moderate, 4 = Satisfied, and 5 = Very satisfied
	Satisfaction compared to other land acquisition policies /X14	1 = Very unsatisfied, 2 = Unsatisfied, 3 = Moderate, 4 = Satisfied, and 5 = Very satisfied

**Table 2:** Respondents’ demographic characteristics.

Variable	Description	Mean	SD
Gender	Male = 1, female = 0	0.631	0.487
Age	Age of respondent (years)	43.776	10.265
Education	Primary school = 1, Middle school = 2, High school = 3, College degree or above = 4	2.100	0.656
Household size	Number of household member (persons)	5.453	1.781
Labor size	Number of the Labor force in a household (persons)	3.107	1.351
Annual family members income	Yuan(CNY)/year	9854	10412
Cultivated land area	The cultivated land area of household (Mu/667 m^2^)	4.702	2.430
Homestead area	Homestead area of household (m^2^)	206.229	99.400

**Table 3:** Validity and reliability for constructs.

Dimensions	Items	Parameters of Significant Test				Item Reliability	Composite Reliability	Convergence Validity
		Estimate	SE.	Est./S.E	*p*-Value	SMC	CR	AVE
EC	X1	0.740	0.037	20.228	***	0.548	0.833	0.556
	X2	0.827	0.03	27.502	***	0.684		
	X3	0.719	0.038	18.743	***	0.517		
	X4	0.691	0.049	14.166	***	0.477		
PE	X5	0.798	0.03	26.446	***	0.637	0.832	0.624
	X6	0.841	0.027	31.519	***	0.707		
	X7	0.727	0.034	21.361	***	0.529		
PPV	X8	0.796	0.026	30.346	***	0.634	0.896	0.683
	X9	0.837	0.023	35.615	***	0.701		
	X10	0.829	0.023	36.684	***	0.687		
	X11	0.842	0.023	36.583	***	0.709		
PS	X12	0.877	0.017	51.017	***	0.769	0.898	0.745
	X13	0.843	0.023	37.312	***	0.711		
	X14	0.869	0.02	44.486	***	0.755		

Note. *** Significant at *p* < 0.001.

**Table 4:** Descriptive statistics and correlations.

Factor	Correlations			
	1	2	3	4
1. ES	(0.746) **			
2. PE	−0.294	(0.790) **		
3. PPV	0.472	−0.642	(0.826) **	
4. PS	0.632	−0.602	0.759	(0.863) **

** *p* < 0.01. Square root of average variance extracted are shown in parentheses, demonstrating discriminant validity. ES: economic status; PE: policy expectation; PV: policy perceived value; PS: policy satisfaction.

**Table 5:** Direct effects of the hypothesized model.

Hypothesis	Path	Estimate	SE.	T	95% Confidence Interval		*p*-Value	Results
					Lower	Upper		
1	ES→PS	0.355	0.053	6.649	0.248	0.462	***	Support
2	PE→PS	−0.201	0.066	−3.020	−0.327	−0.069	0.003	Support
3	PPV→PS	0.463	0.067	6.911	0.332	0.595	***	Support
4	ES→PE	−0.294	0.063	−4.667	−0.412	−0.161	***	Support
5	ES→PPV	0.310	0.058	5.375	0.191	0.423	***	Support
6	PE→PPV	−0.551	0.052	−10.666	−0.652	−0.451	***	Support

Note. Arrow indicates direction of impact; *** Significant at *p* < 0.001.

**Table 6:** Bootstrapping analysis for indirect effects.

Mediating Effects	Point Estimate	Product of Coefficients		*p*-Value	Bootstrap 1000 Times 95% CI			
					Bias-Corrected		Percentile	
		S.E.	Z.		Lower	Upper	Lower	Upper
ES→PE→PS	0.059	0.022	2.632	0.008	0.020	0.108	0.018	0.104
ES→PPV→PS	0.143	0.033	4.317	***	0.088	0.223	0.085	0.218
ES→PExPPV→PS	0.075	0.021	3.513	***	0.041	0.127	0.038	0.121
PE→PPV→PS	−0.255	0.048	−5.305	***	−0.366	−0.17	−0.361	−0.168
ES→PE→PPV	0.162	0.037	4.417	***	0.090	0.236	0.089	0.231

Note. Arrow indicates the direction of impact; *** Significant at *p* < 0.001; 1000 bootstrap samples.
